# Urine trace element disorder along with renal function injury in vitamin D deficient diabetic rats and intervention effect of 1α,25-dihydroxyvitamin D3

**DOI:** 10.3389/fnut.2022.1042558

**Published:** 2022-12-06

**Authors:** Dongxia Wang, Ning Wang, Juan Zhou, Gang Luo, Yanyan Li, Wei Yu, Hongxing Tan, Gang Liu, Jun Wang, Liping Hao

**Affiliations:** ^1^Hubei Key Laboratory of Food Nutrition and Safety, Ministry of Education Key Laboratory of Environment, Department of Nutrition and Food Hygiene, School of Public Health, Tongji Medical College, Huazhong University of Science and Technology, Wuhan, China; ^2^Hebei Key Laboratory of Environment and Human Health, Department of Nutrition and Food Hygiene, School of Public Health, Hebei Medical University, Shijiazhuang, China; ^3^Shenzhen Center for Chronic Disease Control, Shenzhen, China; ^4^Hebei Key Laboratory of Cardiac Injury Repair Mechanism Study, Department of Cardiology, Hebei International Joint Research Center for Structural Heart Disease, The First Hospital of Hebei Medical University, Shijiazhuang, China; ^5^School of Food and Drug, Shenzhen Polytechnic, Shenzhen, China

**Keywords:** 1α, 25-dihydroxyvitamin D3, diabetes, ZDF rats, renal function, urinary Cu, urinary Zn, urinary Se, urinary Mo

## Abstract

**Introduction:**

Trace element metabolism disorders are often secondary to disorders of glucose metabolism in diabetes. Although 1α,25-dihydroxyvitamin D3 [1,25(OH)_2_D_3_] could ameliorate abnormal glucose metabolism in the development of diabetes, the effect on trace element metabolism is unclear. The objective of this study was to evaluate the influence of 1,25(OH)_2_D_3_ on urinary excretions of trace elements in Zucker diabetic fatty (ZDF) rats.

**Methods:**

At 6 weeks of age, male ZDF (*n* = 40) rats were subdivided into four groups: diabetic model (ZDF), low-dose (ZDF + VL, 2 μg/kg⋅bw), middle-dose (ZDF + VM, 8 μg/kg⋅bw) and high-dose (ZDF + VH, 16 μg/kg⋅bw) 1,25(OH)_2_D_3_ groups. Another 10 Zucker lean (ZL) rats served as a control group. All rats were given vitamin D deficient Purina #5008 chow and the intervention groups were given the corresponding dose of 1,25(OH)_2_D_3_ by gavage on alternate days for 7 weeks. Microalbuminuria (MALB) and urinary creatinine concentration were detected by a biochemical autoanalyzer. Urine trace element concentrations were measured using inductively coupled plasma mass spectrometry (ICP-MS) and were corrected by urinary creatinine.

**Results:**

Throughout the intervention phase, MALB, UACR and urinary creatinine levels in the ZDF group were significantly higher than those in the ZL group, and showed a gradual increase with the prolongation of the intervention time. These changes were reversed in a dose-dependent manner after 1,25(OH)_2_D_3_ intervention (*P* < 0.05). Correspondingly, most of the urinary trace element excretions in the ZDF rats were significantly increased compared with the ZL group, and 1,25(OH)_2_D_3_ intervention significantly reduced the urinary copper (Cu), zinc (Zn), selenium (Se) and molybdenum (Mo) levels in the ZDF rats (*P* < 0.05), especially in the medium and high dose groups.

**Conclusion:**

1,25(OH)_2_D_3_ had improvement effects on urinary Cu, Zn, Se, and Mo excretions in ZDF rats, suggesting that it may be related to the reduction of diabetic renal impairment and renal oxidative damage.

## Introduction

Type 2 diabetes mellitus (T2DM) is an endocrine metabolic disease characterized by chronic hyperglycemia and has become a global public health problem that poses a serious threat to human health. According to the latest data published by the International Diabetes Federation, 537 million adults aged 20–79 years have diabetes worldwide in 2021, with a prevalence of 10.5%, of which about 90% are T2DM (1). Among the abovementioned diabetic patients, about 140.9 million are from China, accounting for about a quarter of the global figure. In addition, a nationally representative epidemiological survey showed that the prevalence of diabetes among adults in China was as high as 12.8% in 2017, much higher than 10.9% in 2013 (2). Therefore, the prevention and treatment of T2DM is an important scientific problem that needs to be addressed urgently.

Abnormal glucose metabolism is now recognized as the most fundamental pathophysiological feature in the development of T2DM (3). In addition, the relationship between diabetes and disorders of trace element metabolism has received much attention, largely because most trace elements are involved in glucose metabolism as important components of certain enzymes and hormones (4, 5). It has been shown that trace element metabolism disorders in diabetic patients are often secondary to disorders of glucose metabolism (6). Some studies reported that whole blood levels of zinc, manganese, and chromium were significantly lower while the urinary levels of the same elements were found to be higher in the T2DM patients than in their healthy age-matched counterparts (5). Recent studies found that serum magnesium levels are decreased and serum copper, zinc, and selenium levels are elevated in patients with T2DM (6). In conclusion, we find that the results regarding the association of trace elements and the risk of T2DM are inconsistent.

Vitamin D, an essential fat-soluble vitamin, is mostly converted from 7-dehydrocholesterol in the skin by ultraviolet light exposure. It further undergoes two hydroxylation reactions in the liver and kidney to produce 1α,25-dihydroxyvitamin D3 [1,25(OH)_2_D_3_] before becoming biologically active, and active vitamin D exerts its physiological effects mainly through binding to the Vitamin D Receptor (VDR) (7). Given that VDR is widely distributed in most tissues and cells of the body, vitamin D deficiency is not only associated with disorders of calcium and phosphorus metabolism but also with cancer, autoimmune diseases, metabolic and cardiovascular diseases (diabetes, hypertension, renal diseases, etc.) (8, 9). Evidence from numerous observational studies suggests that vitamin D deficiency is prevalent in patients with T2DM and that the incidence of diabetes is negatively correlated with serum 25(OH)D concentration (10–12). A cross-sectional study based on a Chinese diabetic population showed that 83.5% of patients with T2DM had vitamin D deficiency [serum 25(OH)D concentration < 20 ng/mL], and the prevalence of vitamin D deficiency was more severe in patients with combined diabetic nephropathy than in those without diabetic nephropathy (93.1% vs. 78.9%) (13).

To simulate vitamin D deficiency or insufficiency in T2DM patients, Zucker diabetic fatty (ZDF) rats were fed with vitamin D deficient Purina #5008 chow to establish a vitamin D deficient T2DM animal model. Previous study revealed that vitamin D deficiency accelerated and exacerbated the dysregulation of glucose metabolism in non-obese T2DM rats by increasing insulin resistance and that vitamin D deficiency might be a key factor in the pathogenesis of T2DM (14). The present study further focused on the key role of 1,25(OH)_2_D_3_ supplementation in a vitamin D deficient model of diabetes. No evidence is available regarding the effect of vitamin D supplementation on urinary trace element metabolism in diabetes. The aim of the present study is to clarify whether 1,25(OH)_2_D_3_ supplementation has a protective effect against disorders of trace element excretion in vitamin D-deficient ZDF rats.

## Materials and methods

### Chemicals

1,25(OH)_2_D_3_ (purity ≥ 97%) was obtained from Cayman Chemical (Item No. 71820, USA). Microalbuminuria (MALB) and creatinine assay kits were supplied by Shenzhen Mindray Biomedical Electronics Co., Ltd. (Shenzhen, China), which were used *via* an automatic biochemical analyzer (BS-200, Mindray, Shenzhen, China). All other reagents were analytical grade.

### Animals and diets

Five-week-old male ZDF (*n* = 40) rats and age-matched male Zucker lean (ZL; *n* = 10) rats from Charles River Laboratory (Beijing, China) were maintained on the vitamin D-deficient Purina 5008 chow (500 IU vitamin D3/kg) and tap water *ad libitum*. Animals were kept on a regular 12:12 h light-dark cycle at a controlled temperature (22 ± 2°C) and relative humidity (55 ± 5%). All experimental procedures involving animals complied with the Regulations on the Administration of Laboratory Animals in China and were approved by the Animal Ethical Committee of Tongji Medical College (Approval NO. S432).

### Study design

At the age of 6 weeks, male ZDF rats were subdivided into four groups (*n* = 10): diabetic model (ZDF), low-dose (ZDF + VL, 2 μg/kg⋅bw), middle-dose (ZDF + VM, 8 μg/kg⋅bw), and high-dose (ZDF + VH, 16 μg/kg⋅bw) 1,25(OH)_2_D_3_ groups. Another 10 ZL rats served as a control group. 1,25(OH)_2_D_3_ was dissolved in corn oil and administered by gavage on alternate days for 7 weeks. The vehicle groups (ZL and ZDF) were only gavaged with corn oil.

### Sample collection

At the treatment of 1,4,6,7 weeks, the Zucker rats were housed in metabolic cages and their 24 h urine was dynamically collected at least 3 mL per rat to detect indicators of renal function and minerals. Sodium azide was added to all urine samples at a volume ratio of 10 μL/mL before freezing to protect the urine samples from contamination. When fasting blood glucose (FBG) ≥ 16.7 mmol/L, the model of diabetes was established. At the end of the experiment, the rats were weighed after an overnight fast of 8 h. Blood was collected from the eyes, serum was obtained at least 1 mL per rat from blood samples that has been left at room temperature for 2 h and then centrifuged at 4,000 r/min for 15 min, and the kidneys were removed and weighed. All samples were stored at –80°C for subsequent processing.

### Determination of serum biochemical parameters

FBG was measured by glucose oxidase method according to the manufacturer’s instructions (Biosino Bio-technology and Science Inc., Beijing, China). Serum fasting insulin (FINS) was assayed by double antibody sandwich ELISA using corresponding kit (Millipore Corporation, Billerica, USA). HOMA-IR and HOMA-β were calculated from fasting blood glucose and fasting serum insulin levels with the following formulae:


H⁢O⁢M⁢A-I⁢R=(F⁢I⁢N⁢S×F⁢B⁢G)/22.5;

H⁢O⁢M⁢A-β=(20×F⁢I⁢N⁢S)/(F⁢B⁢G-3.5)⁢(15,16).

Serum triglyceride (TG) concentration was detected by phosphoglyceride oxidase-peroxidase method (GPO-PAP). The levels of total cholesterol (TC), low density lipoprotein cholesterol (LDL-C) and high density lipoprotein cholesterol (HDL-C) in serum were measured by the cholesterol oxidase-peroxidase method (CHOD-PAP). The assay kits of lipid indicators mentioned above were supplied by Biosino Bio-technology and Science Inc. Serum adiponectin was detected using ELISA kit (R&D Systems Inc., Minneapolis, USA). All procedures were carried out in strict accordance with the manufacturer’s protocol.

### Analysis of renal function indicators

24 h urinary collections were taken at 1, 4, 6, and 7 weeks of treatment, respectively. MALB and urinary creatinine concentration were detected by a Mindray BS-200 biochemical autoanalyzer. Subsequently, urea microalbuminuria creatinine ratio (UACR) was calculated *via* the following equation:


U⁢A⁢C⁢R⁢(μ⁢g/m⁢g)=M⁢A⁢L⁢B/(U⁢r⁢i⁢n⁢e⁢c⁢r⁢e⁢a⁢t⁢i⁢n⁢i⁢n⁢e×113).

### Measurement of trace element concentration in urine

Twenty-four hour urine samples were collected at 1, 4, 6, and 7 weeks of treatment and stored at –80^°^C for the dynamic analysis of urinary trace element excretion. Firstly, the urine samples were centrifuged at 4,000 rpm, 4°C for 10 min before being diluted. We mixed 400 μL urine sample and 20 μL of 60% nitric acid solution together and placed them in a refrigerator at 4°C for overnight acidification. Then added 3,580 μL of 1% nitric acid solution to dilute the urine sample 10 times. After centrifuging at 4,000 rpm, 4°C for 10 min, the urine samples were assayed. All samples were measured in triplicate.

Urinary trace element concentrations in the studied samples were detected by inductively coupled plasma mass spectrometer (ICP-MS) at Agilent 7700 (Agilent Technologies, Tokyo, Japan). The obtained data on urinary trace element contents were corrected with urine creatinine concentration and expressed as μg/g UCR.

### Statistical analysis

Data of urine metal concentration were displayed as mean ± SEM or median (IQR). If the data obeyed normal distribution, the statistical significance of comparisons between multiple groups was analyzed by one-way ANOVA, followed by the Least Significant Difference (LSD) multiple range test if the variances were constant, otherwise the Dunnett’s T3 procedure. If the data didn’t follow a normal distribution, we performed a non-parametric (Kruskal-Wallis H) test. All statistical analyses were conducted by SPSS 21.0. *P*-value < 0.05 was considered to show a statistically significant difference.

## Results

### 1,25(OH)_2_D_3_ decreases body weight and kidney index in diabetic rats

**FIGURE 1:**
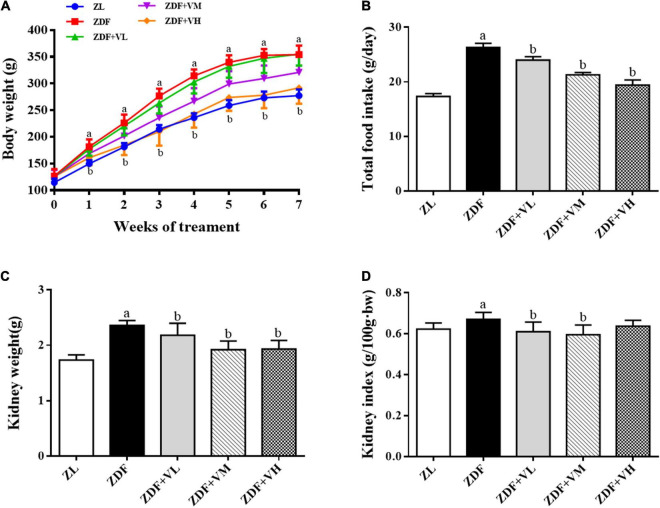
Effects of 1,25(OH)_2_D_3_ on the body weight, food intake, and kidney index in Zucker rats. **(A)** Variation trends of body weight as weeks of 1,25(OH)_2_D_3_ treatment. **(B)** Total food intake. **(C)** Kidney weight. **(D)** Kidney index. ZL, Zucker lean group; ZDF, Zucker diabetic fatty group; ZDF + VL, ZDF + low-dose 1,25(OH)_2_D_3_ group (2 μg/kg⋅bw); ZDF + VM, ZDF + middle-dose 1,25(OH)_2_D_3_ group (8 μg/kg⋅bw); ZDF + VH, ZDF + high-dose 1,25(OH)_2_D_3_ group (16 μg/kg⋅bw). Data are presented as mean ± SD (*n* ≥ 7). ^a^*P* < 0.05 vs. the ZL group, ^b^*P* < 0.05 vs. the ZDF group.

ZDF rats were fed with Purina #5008 chow for 7 weeks to establish a spontaneous T2DM rat model, and 1,25(OH)_2_D_3_ was administered at the same time. As shown in Figure 1, there was no significant difference in the initial body weight (week 0) of the rats in each group. With the prolongation of the intervention time, the body weight of the rats in each group gradually increased. From the first week of intervention to the end of the experiment, the body weight of the rats in the diabetes group (ZDF) was significantly higher than that in the control group (ZL). While the body weight of the rats in the high-dose 1,25(OH)_2_D_3_ intervention group (ZDF + VH) was significantly lower than that in the ZDF group (*P* < 0.05). At the 7th week of intervention, the body weight of the rats in the ZDF group increased by 28% compared with the ZL group, while compared with the ZDF group, the figure decreased by 18% in the ZDF + VH group (*P* < 0.05). Meanwhile, the results showed that the weight of kidneys and kidney index in the ZDF group were significantly increased compared with those in the ZL group, and the 1,25(OH)_2_D_3_ dose groups effectively reversed the above changes.

### 1,25(OH)_2_D_3_ improves insulin sensitivity and lipid metabolism in diabetic rats

As shown in Table 1, the levels of fasting blood glucose, serum insulin, and HOMA-IR in the ZDF group were significantly higher than those in the ZL group. Although the serum insulin in each 1,25(OH)_2_D_3_ intervention group was at a high level, the fasting blood glucose and HOMA-IR in each dose group were significantly lower than those in the ZDF group, and the function of HOMA-β was significantly enhanced (*P* < 0.05). It indicated that 1,25(OH)_2_D_3_ played an important role in increasing insulin sensitivity, and the improvement effect was most promising in the middle and high dose groups.

**TABLE 1:** Effects of 1,25(OH)_2_D_3_ on insulin sensitivity and parameters of lipid metabolism in Zucker rats.

Groups	ZL	ZDF	ZDF + VL	ZDF + VM	ZDF + VH
FBG (mmol/L)	4.39 ± 0.40	17.26 ± 3.17^a^	10.05 ± 2.43^b^	6.34 ± 0.36^b^	6.77 ± 0.43^b^
FINS (mIU/L)	14.48 ± 5.80	245.97 ± 69.79^a^	439.95 ± 190.94	334.39 ± 99.56	225.89 ± 65.58
HOMA-IR	3.02 ± 1.59	205.08 ± 46.99^a^	213.18 ± 55.88	77.05 ± 13.66^b^	69.73 ± 19.94^b^
HOMA-β	298.48 ± 23.25	364.83 ± 195.55	1121.19 ± 680.80^b^	2456.62 ± 706.73^b^	1381.12 ± 325.43^b^
Serum TG (mmol/L)	2.80 ± 0.83	11.12 ± 4.05^a^	12.50 ± 4.21	6.97 ± 1.50^b^	5.75 ± 1.28^b^
Serum TC (mmol/L)	3.02 ± 0.13	4.47 ± 0.48^a^	4.25 ± 0.70	4.13 ± 0.52	3.99 ± 0.44
Serum HDL-C (mmol/L)	0.43 ± 0.13	0.40 ± 0.10	0.44 ± 0.16	0.24 ± 0.05^b^	0.26 ± 0.07^b^
Serum LDL-C (mmol/L)	1.19 ± 0.12	1.79 ± 0.23^a^	1.69 ± 0.51	1.66 ± 0.30	1.59 ± 0.49
Serum adiponectin (ng/mL)	7.46 ± 1.15	5.52 ± 1.12^a^	5.61 ± 1.42	8.13 ± 1.62^b^	9.29 ± 1.92^b^

ZL, Zucker lean group; ZDF, Zucker diabetic fatty group. ZDF + VL, ZDF + low-dose 1,25(OH)_2_D_3_ group (2 μg/kg⋅bw); ZDF + VM, ZDF + middle-dose 1,25(OH)_2_D_3_ group (8 μg/kg⋅bw); ZDF + VH, ZDF + high-dose 1,25(OH)_2_D_3_ group (16 μg/kg⋅bw). Data are presented as the mean ± SD (*n* ≥ 4). ^a^*P* < 0.05 vs. the ZL group, ^b^*P* < 0.05 vs. the ZDF group using one way ANOVA tests.

Compared with the ZL group, the serum TG, TC, and LDL-C levels of the ZDF group were increased by 297, 48, and 50%, respectively (*P* < 0.05). On the contrary, the serum TG levels of the ZDF + VM and ZDF + VH groups were decreased by 37 and 48% than the ZDF group (*P* < 0.05), meanwhile the serum TC and LDL-C concentrations showed a dose-dependent decrease. Adiponectin is an adipokine produced by adipose tissue, which is involved in glucose and lipid metabolism and inflammatory response. The serum adiponectin level in the ZDF group was 26% lower than that in the ZL group (*P* < 0.05). However, the serum adiponectin levels in the ZDF + VM and ZDF + VH groups were increased by 47 and 68%, respectively, compared with the ZDF group (*P* < 0.05). The above results indicate that medium and high doses of 1,25(OH)_2_D_3_ supplementation can improve the abnormal lipid metabolism in spontaneous diabetic rats, and its lipid-lowering effect is mainly manifested by a significant reduction in TG levels and an increase in adiponectin levels.

### 1,25(OH)_2_D_3_ alleviates renal function damage in diabetic rats

**FIGURE 2:**
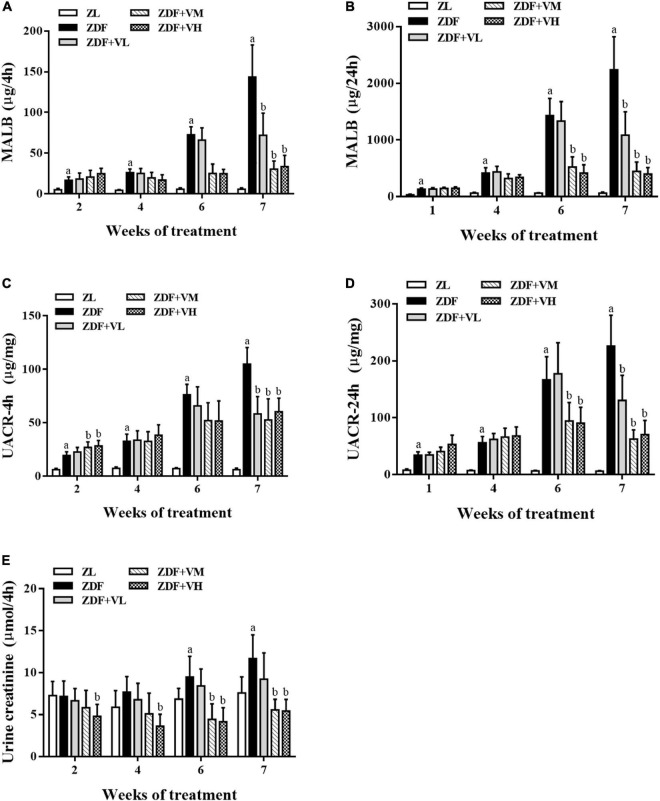
Variation trend of renal function indicators as weeks of 1,25(OH)_2_D_3_ treatment. **(A,B)** Four hour-MALB and 24 h-MALB contents. **(C,D)** Four hour-UACR and 24 h-UACR contents. **(E)** Four hour-Urine creatinine content. ZL, Zucker lean group; ZDF, Zucker diabetic fatty group; ZDF + VL, ZDF + low-dose 1,25(OH)_2_D_3_ group (2 μg/kg⋅bw); ZDF + VM, ZDF + middle-dose 1,25(OH)_2_D_3_ group (8 μg/kg⋅bw); ZDF + VH, ZDF + high-dose 1,25(OH)_2_D_3_ group (16 μg/kg⋅bw). Data are presented as mean ± SD (n ≥ 4). ^a^*P* < 0.05 vs. the ZL group, ^b^*P* < 0.05 vs. the ZDF group.

To investigate whether 1,25(OH)_2_D_3_ has a nephroprotective effect on ZDF rats, we used UACR and urinary creatinine levels as indicators to evaluate the progress of DN. As shown in Figure 2, the MALB, UACR, and urine creatinine contents in the ZDF group were significantly higher than those in the ZL group during the whole intervention period, and the gap was gradually widened with the extension of the intervention time. These changes were reversed in a dose-dependent manner after 1,25(OH)_2_D_3_ intervention (*P* < 0.05).

### Effect of 1,25(OH)_2_D_3_ on the disturbance of urinary trace element metabolism in diabetic rats

#### Effect of 1,25(OH)_2_D_3_ on urinary trace elements in spontaneously diabetic rats at 1st week of intervention

In this study, 23 trace element concentrations in urine were dynamically determined by ICP-MS and corrected with urinary creatinine concentrations in μg/g UCR, except Tl, Sb, Cd, Sn, and U which were in ng/g UCR. As seen in Table 2, at 1 week of intervention, urinary levels of Cu, Zn, Se, Mo, As, W, Ti, Co, Cr, Ni, Sr, and Cd were significantly increased in the ZDF group of rats compared to the ZL group, and 1,25(OH)_2_D_3_ intervention increased the levels of Se, Tl, Ti, Cr, Al, Mn, Sr, Cd, and Ba in urine compared with the ZDF group (*P* < 0.05).

**TABLE 2:** Effects of 1,25(OH)_2_D_3_ on urine metal concentration of Zucker rats in the first week of treatment.

Metals (μg/g UCR)	ZL	ZDF	ZDF + VL	ZDF + VM	ZDF + VH
Cu	184.66 ± 4.98	243.81 ± 7.83^a^	235.21 ± 10.60	223.87 ± 10.49	240.28 ± 13.10
Zn	143.93 ± 23.21	245.87 ± 13.42^a^	262.43 ± 19.72	231.91 ± 7.81	240.25 ± 25.49
Se	7.24 ± 0.18	13.18 ± 0.67^a^	17.03 ± 0.59^b^	16.07 ± 0.88	18.21 ± 1.27^b^
Mo	261.38 ± 5.96	411.78 ± 10.95^a^	406.63 ± 12.99	366.08 ± 27.50	375.01 ± 19.52
As	13.72 ± 0.43	17.43 ± 0.45^a^	17.33 ± 0.68	17.57 ± 0.96	18.51 ± 0.60
Rb	1084.69 ± 48.47	2081.95 ± 57.83	2425.84 ± 56.45	2420.95 ± 85.33	2519.00 ± 93.90
V	1.19 ± 0.10	1.81 ± 0.17	1.98 ± 0.21	1.99 ± 0.24	2.31 ± 0.20
W	0.47 ± 0.02	0.59 ± 0.02^a^	0.58 ± 0.03	0.53 ± 0.03	0.60 ± 0.03
Tl	124.98 ± 11.40	269.56 ± 9.72	350.71 ± 10.55	359.09 ± 18.41	373.69 ± 15.00^b^
Ti	4.07 (2.25,10.63)	38.98 (30.55,44.30)^a^	68.39 (49.09,77.64)^b^	85.09 (66.89,104.78)^b^	100.73 (79.98,122.21)^b^
Co	2.67 (2.16,2.73)	4.54 (3.97, 4.91)^a^	5.44 (4.70, 5.72)	4.34 (4.15, 5.18)	5.03 (4.14, 6.16)
Cr	0.81 (0.56, 0.95)	1.81 (1.37, 2.50)^a^	2.72 (2.05, 3.58)^b^	2.34 (2.04, 2.86)	2.57 (2.01, 3.71)^b^
Ni	8.59 ± 1.62	21.23 ± 2.19^a^	25.62 ± 3.76	23.62 ± 2.02	25.84 ± 2.50
Sb	116.02 ± 9.58	127.94 ± 5.35	112.14 ± 7.09	116.71 ± 12.61	115.42 ± 6.31
Al	13.39 ± 2.30	21.09 ± 1.60	24.39 ± 3.82	24.93 ± 2.69	30.86 ± 3.10^b^
Fe	32.51 ± 8.11	67.53 ± 5.85	86.59 ± 13.06	96.44 ± 9.82	90.59 ± 15.47
Mn	11.36 ± 2.28	32.76 ± 5.64	67.74 ± 17.01	138.94 ± 10.38^b^	116.47 ± 27.24
Sr	91.06 ± 6.55	182.34 ± 13.14^a^	254.26 ± 18.75	329.87 ± 21.10^b^	356.21 ± 24.34^b^
Cd	180.81 ± 7.34	244.99 ± 9.17^a^	277.64 ± 14.74	299.18 ± 22.50^b^	302.94 ± 17.47^b^
Sn	169.89 ± 20.65	123.69 ± 11.00	101.14 ± 10.18	111.74 ± 18.96	95.51 ± 7.48
Ba	6.74 (5.86, 9.34)	8.85 (6.87, 9.29)	11.62 (8.19, 17.08)	18.68 (15.40, 21.96)^b^	18.35 (12.31, 25.70)^b^
Pb	0.40 ± 0.05	0.37 ± 0.07	0.42 ± 0.08	0.32 ± 0.07	0.21 ± 0.03
U	263.85 ± 34.25	134.44 ± 13.09	100.12 ± 8.90	110.48 ± 8.09	108.60 ± 11.88

ZL, Zucker lean group; ZDF, Zucker diabetic fatty group. ZDF + VL, ZDF + low-dose 1,25(OH)_2_D_3_ group (2 μg/kg⋅bw); ZDF + VM, ZDF + middle-dose 1,25(OH)_2_D_3_ group (8 μg/kg⋅bw); ZDF + VH, ZDF + high-dose 1,25(OH)_2_D_3_ group (16 μg/kg⋅bw). The unite of Tl, Sb, Cd, Sn and U was expressed as ng/g UCR. Data are presented as mean ± SEM or median (IQR) (*n* ≥ 8). ^a^*P* < 0.05 vs. the ZL group, ^b^*P* < 0.05 vs. the ZDF group using one way ANOVA tests.

#### Effect of 1,25(OH)_2_D_3_ on urinary trace elements in spontaneously diabetic rats at 4th week of intervention

As seen from Table 3, after 4 weeks of intervention, the urinary levels of Zn, Se, Mo, As, Rb, V, W, Tl, Ti, Co, Cr, Ni, Sb, Al, Fe, Mn, Cd, Ba, and U in the ZDF group of rats were significantly increased compared to the ZL group. And the urinary levels of Se, Mo, As, Rb, W, Ni, Sb, Sn, and U after 1,25(OH)_2_D_3_ intervention were significantly decreased compared to the ZDF group, while Sr and Ba levels were increased compared to the ZDF group (*P* < 0.05).

**TABLE 3:** Effects of 1,25(OH)_2_D_3_ on urine metal concentration of Zucker rats in the fourth week of treatment.

Metals (μg/g UCR)	ZL	ZDF	ZDF + VL	ZDF + VM	ZDF + VH
Cu	124.73 ± 6.21	175.24 ± 11.69	227.33 ± 15.93	180.33 ± 16.62	155.38 ± 12.27
Zn	78.98 ± 4.09	323.70 ± 13.83^a^	324.78 ± 18.42	251.30 ± 20.77	210.64 ± 25.16
Se	7.23 ± 0.48	17.27 ± 1.10^a^	18.13 ± 1.23	14.58 ± 0.64^b^	13.19 ± 0.44^b^
Mo	179.97 ± 9.65	246.72 ± 12.79^a^	280.37 ± 17.30	214.16 ± 11.76	178.17 ± 7.08^b^
As	10.26 ± 0.42	14.22 ± 0.67^a^	16.14 ± 0.79^b^	14.13 ± 0.72	12.35 ± 0.38^b^
Rb	758.61 ± 39.41	1658.90 ± 65.63^a^	1756.48 ± 89.96	1507.59 ± 76.31	1357.63 ± 62.20^b^
V	0.81 ± 0.07	1.77 ± 0.07^a^	2.15 ± 0.23	1.64 ± 0.12	1.64 ± 0.13
W	0.30 ± 0.01	0.44 ± 0.02^a^	0.48 ± 0.02	0.42 ± 0.02	0.37 ± 0.01^b^
Tl	79.94 ± 7.82	247.06 ± 11.55^a^	278.67 ± 12.78	235.41 ± 10.79	226.95 ± 9.71
Ti	15.86 ± 1.89	68.61 ± 4.20^a^	95.10 ± 6.37	74.15 ± 4.33	63.86 ± 3.42
Co	1.61 ± 0.13	2.71 ± 0.16^a^	3.72 ± 0.27	2.95 ± 0.24	2.43 ± 0.14
Cr	1.01 (0.81,1.45)	3.64 (2.85, 4.77)^a^	2.99 (2.04, 4.19)	1.78 (1.54, 3.07)	2.39 (1.63, 2.96)
Ni	7.05 (5.76, 7.79)	22.86 (22.13, 25.07)^a^	23.92 (17.44, 32.54)	17.17 (13.62, 20.68)	15.78 (13.17, 19.29)^b^
Sb	64.24 ± 4.06	122.21 ± 5.96^a^	129.47 ± 10.90	88.16 ± 6.85^b^	83.05 ± 6.71^b^
Al	16.28 ± 1.15	47.98 ± 3.08^a^	45.80 ± 6.62	40.69 ± 3.66	41.22 ± 4.70
Fe	37.00 (30.06, 42.73)	160.15 (130.83, 187.32)^a^	157.40 (118.70, 234.62)	143.06 (103.51, 232.23)	130.34 (104.25, 301.02)
Mn	7.82 ± 0.80	136.11 ± 14.05^a^	176.39 ± 20.69	191.75 ± 25.01	178.98 ± 28.79
Sr	35.38 ± 2.08	82.67 ± 3.98	240.48 ± 23.86^b^	223.33 ± 19.04^b^	171.14 ± 15.84
Cd	107.04 ± 5.34	200.58 ± 12.13^a^	262.67 ± 20.53	225.79 ± 20.18	213.79 ± 20.61
Sn	203.23 ± 16.88	193.63 ± 19.15	141.30 ± 13.97	97.07 ± 3.83^b^	85.28 ± 6.63^b^
Ba	3.93 ± 0.43	11.13 ± 0.40^a^	19.86 ± 2.24^b^	20.48 ± 1.56^b^	19.81 ± 2.05^b^
Pb	0.20 (0.16, 0.30)	0.46 (0.24, 0.65)	0.26 (0.16, 0.90)	0.19 (0.14, 0.49)	0.33 (0.11, 0.63)
U	201.42 ± 13.23	270.76 ± 19.36^a^	160.84 ± 18.68^b^	173.86 ± 17.90^b^	186.53 ± 24.42^b^

ZL, Zucker lean group; ZDF, Zucker diabetic fatty group. ZDF + VL, ZDF + low-dose 1,25(OH)_2_D_3_ group (2 μg/kg⋅bw); ZDF + VM, ZDF + middle-dose 1,25(OH)_2_D_3_ group (8 μg/kg⋅bw); ZDF + VH, ZDF + high-dose 1,25(OH)_2_D_3_ group (16 μg/kg⋅bw). The unite of Tl, Sb, Cd, Sn, and U was expressed as ng/g UCR. Data are presented as mean ± SEM or median (IQR) (*n* ≥ 9). ^a^*P* < 0.05 vs. the ZL group, ^b^*P* < 0.05 vs. the ZDF group using one way ANOVA tests.

#### Effect of 1,25(OH)_2_D_3_ on urinary trace elements in spontaneously diabetic rats at 6th week of intervention

As seen from Table 4, after 6 weeks of intervention, the urinary levels of Cu, Zn, Se, Mo, As, Rb, V, W, Tl, Ti, Co, Cr, Ni, Sb, Al, Fe, Mn, Cd, Sn, Pb, and U in the ZDF group of rats were significantly increased compared to the ZL group. And the urinary levels of Cu, Zn, Mo, As, Rb, Cr, and U after 1,25(OH)_2_D_3_ intervention decreased compared with the ZDF group, while Sr level increased compared with the ZDF group (*P* < 0.05).

**TABLE 4:** Effects of 1,25(OH)_2_D_3_ on urine metal concentration of Zucker rats in the sixth week of treatment.

Metals (μg/g UCR)	ZL	ZDF	ZDF + VL	ZDF + VM	ZDF + VH
Cu	109.08 ± 5.42	199.31 ± 14.34^a^	164.56 ± 11.00^b^	142.63 ± 8.53^b^	166.97 ± 6.29^b^
Zn	62.54 ± 2.22	393.50 ± 27.54^a^	313.71 ± 29.80	250.58 ± 23.82^b^	257.75 ± 26.79^b^
Se	6.26 ± 0.31	22.07 ± 2.02^a^	18.83 ± 1.75	14.79 ± 0.69	16.03 ± 0.80
Mo	148.06 ± 8.49	233.96 ± 10.46^a^	207.62 ± 10.57	178.25 ± 6.12^b^	166.84 ± 12.83^b^
As	9.26 ± 0.39	15.88 ± 0.92^a^	15.58 ± 0.81	12.88 ± 0.54^b^	12.11 ± 0.85^b^
Rb	668.60 ± 25.85	1773.86 ± 84.74^a^	1646.44 ± 78.37	1359.78 ± 51.40	1161.21 ± 104.14^b^
V	0.85 (0.79, 1.04)	2.25 (1.61, 3.25)^a^	2.01 (1.49, 2.56)	1.63 (1.51, 2.08)	2.10 (1.48, 2.76)
W	0.23 ± 0.01	0.47 ± 0.04^a^	0.45 ± 0.03	0.37 ± 0.01	0.35 ± 0.04
Tl	56.24 ± 4.08	261.25 ± 15.29^a^	245.89 ± 12.34	225.46 ± 12.09	203.13 ± 15.32
Ti	12.42 ± 1.15	73.87 ± 5.19^a^	90.49 ± 8.03	74.94 ± 3.53	72.49 ± 7.36
Co	1.62 ± 0.05	3.12 ± 0.25^a^	3.16 ± 0.29	2.69 ± 0.09	2.64 ± 0.30
Cr	0.71 ± 0.04	4.84 ± 0.76^a^	3.22 ± 0.48	1.83 ± 0.25^b^	2.04 ± 0.22
Ni	7.87 ± 0.65	33.84 ± 3.91^a^	29.32 ± 4.40	19.95 ± 1.83	21.00 ± 3.89
Sb	33.89 ± 1.85	118.03 ± 11.32^a^	90.52 ± 3.30	80.52 ± 5.90	84.91 ± 8.67
Al	13.09 ± 0.99	67.87 ± 9.30^a^	53.41 ± 9.35	38.68 ± 4.85	43.17 ± 7.43
Fe	37.26 (32.80, 49.50)	194.24 (142.57, 285.08)^a^	180.67 (125.17, 217.62)	153.23 (106.00, 355.21)	263.92 (111.32, 390.51)
Mn	4.62 ± 0.48	238.38 ± 29.77^a^	258.95 ± 45.63	279.53 ± 42.50	320.14 ± 68.20
Sr	29.53 ± 1.63	95.48 ± 4.76	197.15 ± 16.00	218.55 ± 16.94^b^	247.38 ± 24.49^b^
Cd	76.57 ± 4.04	201.91 ± 16.74^a^	235.17 ± 25.16	238.33 ± 19.34	258.73 ± 38.69
Sn	32.72 ± 1.40	165.66 ± 26.52^a^	94.59 ± 12.36	76.02 ± 6.63	92.63 ± 16.55
Ba	2.51 ± 0.23	12.89 ± 1.31	18.37 ± 1.45	20.30 ± 1.34	20.12 ± 2.18
Pb	0.13 ± 0.01	0.84 ± 0.22^a^	0.41 ± 0.11	0.33 ± 0.09	0.38 ± 0.07
U	156.91 ± 10.00	261.99 ± 22.49^a^	148.10 ± 18.01^b^	201.41 ± 35.95	226.30 ± 32.70

ZL, Zucker lean group; ZDF, Zucker diabetic fatty group. ZDF + VL, ZDF + low-dose 1,25(OH)_2_D_3_ group (2 μg/kg⋅bw); ZDF + VM, ZDF + middle-dose 1,25(OH)_2_D_3_ group (8 μg/kg⋅bw); ZDF + VH, ZDF + high-dose 1,25(OH)_2_D_3_ group (16 μg/kg⋅bw). The unite of Tl, Sb, Cd, Sn, and U was expressed as ng/g UCR. Data are presented as mean ± SEM or median (IQR) (*n* ≥ 9). ^a^*P* < 0.05 vs. the ZL group, ^b^*P* < 0.05 vs. the ZDF group using one way ANOVA tests.

#### Effect of 1,25(OH)_2_D_3_ on urinary trace elements in spontaneously diabetic rats at 7th week of intervention

As can be seen from Table 5, after 7 weeks of intervention, the urinary levels of Cu, Zn, Se, Mo, As, Rb, V, W, Tl, Ti, Co, Cr, Ni, Sb, Al, Fe, Mn, Cd, and Sn in the ZDF group of rats were significantly increased compared to the ZL group, and the urinary levels of Cu, Zn, Se, Mo after 1,25(OH)_2_D_3_ intervention decreased compared to the ZDF group, while Sr level increased compared to the ZDF group after 1,25(OH)_2_D_3_ intervention (*P* < 0.05).

**TABLE 5:** Effects of 1,25(OH)_2_D_3_ on urine metal concentration of Zucker rats in the seventh week of treatment.

Metals (μg/g UCR)	ZL	ZDF	ZDF + VL	ZDF + VM	ZDF + VH
Cu	83.46 ± 4.68	165.69 ± 6.73^a^	129.57 ± 12.55^b^	115.86 ± 8.45^b^	138.64 ± 6.27^b^
Zn	58.34 ± 5.06	364.47 ± 26.67^a^	289.48 ± 34.78	182.51 ± 17.71^b^	176.57 ± 9.78^b^
Se	6.24 ± 0.31	23.44 ± 2.34^a^	18.68 ± 1.98	12.46 ± 0.63^b^	12.97 ± 0.58^b^
Mo	134.93 ± 6.24	211.04 ± 6.79^a^	204.13 ± 12.74	179.93 ± 5.14^b^	176.77 ± 10.52^b^
As	8.03 ± 0.30	14.30 ± 0.94^a^	13.40 ± 0.80	11.49 ± 0.39	12.15 ± 0.34
Rb	593.46 ± 20.74	1689.40 ± 58.11^a^	1551.81 ± 106.79	1503.00 ± 50.90	1520.98 ± 76.37
V	0.83 ± 0.07	1.89 ± 0.18^a^	1.58 ± 0.12	1.71 ± 0.19	2.29 ± 0.21
W	0.21 ± 0.01	0.41 ± 0.03^a^	0.36 ± 0.03	0.35 ± 0.01	0.36 ± 0.02
Tl	42.80 ± 2.83	244.11 ± 8.85^a^	247.83 ± 12.76	219.54 ± 3.50	223.77 ± 12.35
Ti	6.50 ± 1.27	63.26 ± 4.70^a^	65.23 ± 5.03	50.09 ± 2.19	50.38 ± 2.63
Co	1.12 ± 0.07	2.63 ± 0.20^a^	2.72 ± 0.28	2.17 ± 0.12	2.31 ± 0.04
Cr	0.65 ± 0.06	3.67 ± 0.74^a^	1.83 ± 0.37	1.25 ± 0.14	2.42 ± 0.52
Ni	5.60 (4.71, 7.29)	24.44 (18.16, 31.77)^a^	16.58 (13.18, 21.17)	12.87 (11.31, 18.29)	17.94 (15.56, 21.99)
Sb	28.43 ± 1.66	75.25 ± 9.27^a^	74.19 ± 7.07	69.79 ± 5.04	83.22 ± 6.63
Al	8.79 ± 0.94	48.90 ± 5.65^a^	35.03 ± 7.34	31.14 ± 5.84	39.99 ± 5.72
Fe	26.08 ± 3.76	151.82 ± 13.84^a^	140.00 ± 11.97	143.16 ± 15.32	223.45 ± 23.37
Mn	4.47 ± 0.79	189.60 ± 17.49^a^	200.76 ± 23.82	169.96 ± 14.34	227.92 ± 22.15
Sr	25.64 ± 1.95	93.12 ± 5.09	163.37 ± 6.59^b^	139.96 ± 13.47	143.47 ± 10.97
Cd	68.60 ± 2.47	169.88 ± 9.64^a^	190.23 ± 12.46	176.55 ± 13.37	187.58 ± 9.15
Sn	23.10 (20.84, 24.77)	55.44 (37.00, 80.77)^a^	63.84 (45.82, 104.49)	48.76 (43.89, 55.70)	52.79 (46.52, 66.67)
Ba	1.75 ± 0.18	10.46 ± 0.73	15.26 ± 0.93	14.22 ± 1.21	15.05 ± 1.58
Pb	0.11 (0.08, 0.17)	0.25 (0.16, 0.49)	0.12 (0.10, 0.30)	0.11 (0.09, 0.17)	0.29 (0.16, 0.78)
U	124.09 ± 13.98	172.70 ± 20.56	111.73 ± 8.90	157.34 ± 15.67	191.45 ± 24.99

ZL, Zucker lean group; ZDF, Zucker diabetic fatty group. ZDF + VL, ZDF + low-dose 1,25(OH)_2_D_3_ group (2 μg/kg⋅bw); ZDF + VM, ZDF + middle-dose 1,25(OH)_2_D_3_ group (8 μg/kg⋅bw); ZDF + VH, ZDF + high-dose 1,25(OH)_2_D_3_ group (16 μg/kg⋅bw). The unite of Tl, Sb, Cd, Sn and U was expressed as ng/g UCR. Data are presented as mean ± SEM or median (IQR) (*n* ≥ 9). ^a^*P* < 0.05 vs. the ZL group, ^b^*P* < 0.05 vs. the ZDF group using one way ANOVA tests.

#### Dynamic analysis of copper, zinc, selenium, and molybdenum levels in the urine of spontaneously diabetic rats and the effect of 1,25(OH)_2_D_3_ intervention

**FIGURE 3:**
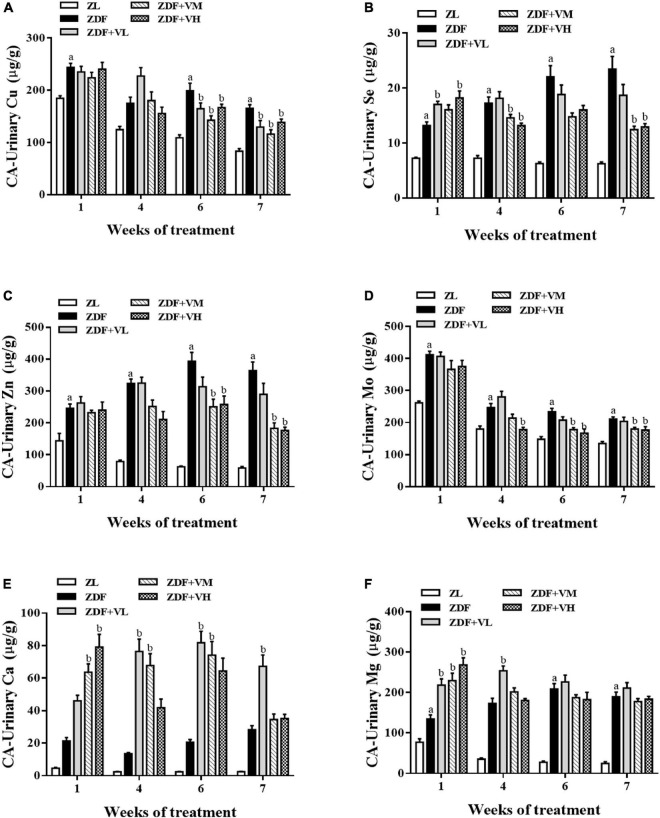
Effect of 1,25(OH)_2_D_3_ on urinary mineral levels in Zucker rats. **(A)** Urinary Cu/UCR. **(B)** Urinary Se/UCR. **(C)** Urinary Zn/UCR. **(D)** Urinary Mo/UCR. **(E)** Urinary Ca/UCR. **(F)** Urinary Mg/UCR. ZL, Zucker lean group; ZDF, Zucker diabetic fatty group; ZDF + VL, ZDF + low-dose 1,25(OH)_2_D_3_ group (2 μg/kg⋅bw); ZDF + VM, ZDF + middle-dose 1,25(OH)_2_D_3_ group (8 μg/kg⋅bw); ZDF + VH, ZDF + high-dose 1,25(OH)_2_D_3_ group (16 μg/kg⋅bw). Data are presented as mean ± SEM (*n* ≥ 9). ^a^*P* < 0.05 vs. the ZL group, ^b^*P* < 0.05 vs. the ZDF group.

From Tables 2–5, it was found that the trend of Cu, Zn, Se, and Mo levels in the urine of spontaneously diabetic rats after 1,25(OH)_2_D_3_ intervention was the most obvious. These four trace elements were thus plotted to visualize their dynamic changes with the extension of the intervention time. As shown in Figures 3A–D, the urinary levels of Cu, Zn, Se, and Mo in the ZDF group increased significantly (*P* < 0.05) compared with the ZL group at 1st week of intervention. At 4th week of intervention, the urinary levels of Cu, Zn, Se, and Mo in rats in each dose group of 1,25(OH)_2_D_3_ showed a tendency to decrease compared with the ZDF group. And with the extension of the intervention time, the differences of the above four urinary trace elements levels in the rats of ZDF + VM and ZDF + VH groups gradually increased compared with the ZDF group, which to some extent coincided with the trend of urine volume. Additionally, we also measured the urinary levels of calcium (Ca) and magnesium (Mg). As displayed in Figures 3E,F, the urinary levels of Ca and Mg in the ZDF group increased compared with the ZL group during the whole intervention, but the differences were not significant for urinary Ca. Relatively, the urinary levels of Ca and Mg in rats in each dose group of 1,25(OH)_2_D_3_ maintained high levels even upper than the ZDF group.

## Discussion

The present study provides evidence that 1,25(OH)_2_D_3_ improves kidney index, insulin sensitivity, abnormal glucose and lipid metabolism, renal function injury and urine trace element metabolism disorder in spontaneous diabetic rats. We found that spontaneous diabetes could lead to renal function damage by increasing MALB, UACR, urine creatinine contents, and exacerbate the disorder of most trace elements in urine. As predicted, we showed that 1,25(OH)_2_D_3_ treatment significantly attenuated renal damage and decreased urine Cu, Zn, Se, and Mo contents. However, given that there are few studies on vitamin D reducing urinary trace elements in diabetic patients or models, more studies are needed to confirm this conclusion in the future.

Evidence has reported that MALB is a marker of early kidney damage in DN. In our study, the continuous increase of MALB content confirmed the occurrence and development of renal dysfunction in spontaneous diabetic rats, accompanied by the continuous increase of UACR and urinary creatinine content. Importantly, treatment with 1,25(OH)_2_D_3_ decreased the impairment of renal function. Similarly, 1,25(OH)_2_D_3_ has been reported to ameliorate renal dysfunction in rats with chronic renal failure (17) or may contribute to delay the deterioration in glomerular function and reduce the occurrence of ESRD in patients with diabetes (18). Besides, our previous research has revealed the positive effect of 1,25(OH)_2_D_3_ on renal morphological, pathological changes and oxidative stress damage (19). Considering that trace elements are closely related to diabetes, the levels of trace elements in urine were further detected. It was found that the metabolism of trace elements in the urine of spontaneous diabetic rats was disordered, and the intervention of 1,25(OH)_2_D_3_ could significantly reverse the urinary Cu, Zn, Se, and Mo contents of ZDF rats. The results also suggested that the disturbance of trace element metabolism in ZDF rats might be related to kidney damage.

Both a cross-sectional study and a multisite, multiethnic cohort study indicated that increased urinary excretion of zinc was associated with elevated risk of diabetes (20, 21). Some scholars have studied the effect of STZ-induced insulin-dependent diabetes on urinary excretion of zinc, copper and iron, and found that the onset of diabetes symptoms was associated with a rapid and sustained increase in the daily excretion of these three trace elements in urine, which was significantly reduced by insulin treatment (22). Combined with our study, it can be indicated that 1,25(OH)_2_D_3_ intervention can achieve the efficacy of insulin therapy to a certain extent. Another study indicated that diabetes can alter levels of copper, zinc, magnesium, and lipid peroxidation, and it also highlighted that disturbance in mineral metabolism was more pronounced in diabetic patients with specific complications (23). Gong later suggested that an imbalance in copper homeostasis may be a key event in triggering the development of diabetic nephropathy (24). Our present study further found that 1,25(OH)_2_D_3_ may reduce diabetic kidney injury by restoring urinary copper levels.

Currently, urinary trace element levels are expressed in different ways. Data from a study in Northeast China showed an increase in urinary Zn levels, a decrease in the Zn/Cu ratio, and a decrease in urinary Se levels in patients with T2DM (25). A cross-sectional study has indicated significantly higher urinary Zn levels and no significant changes in urinary Cu, Mo, and Se levels in diabetic patients as compared to healthy subjects (26). However, it is worth noting that these studies present trace element concentrations rather than contents. In a STZ-induced mild diabetic rat model, 24-h urinary excretion of zinc, copper, iron, calcium, and magnesium was found to be positively correlated with urine volume (27), whose daily mineral excretion was determined by multiplying the daily urine volume by the concentration of each mineral in the urine. In most studies, however, such results are calculated by the ratio of the concentration of minerals in urine to the concentration of urinary creatinine. Our research used the latter method, while suggesting that the excretions of minerals in urine were proportional to the amount of urine. The reason could be that hyperglycemia may cause hyperosmolar urine or impair tubular reabsorption of trace elements which may further hamper tubular function and lead to hyperfiltration.

In a STZ-induced diabetic rat model, it showed increased levels of Fe and Cu and decreased levels of Zn and Mg in liver and kidney tissues, decreased contents of 24 h urinary Cu and increased contents of 24 h urinary Zn and Mg (28). This study suggests that impaired trace element metabolism may be associated with disturbances in oxidative homeostasis in liver and kidney tissues. More importantly, vitamin D supplementation modulated blood and tissue zinc concentrations, hepatic glutathione, and blood biochemical parameters in diabetic rats (29). However, studies on whether vitamin D supplementation modulates urinary trace elements in diabetic rats are relatively rare. Compared with previous studies, this study used ICP-MS to dynamically detect the concentrations of more than 20 trace elements in urine, and at the same time normalized and corrected the concentration of urine creatinine. It has made a comprehensive and systematic evaluation of the preventive effect of 1,25(OH)_2_D_3_ on urinary trace elements disorders in diabetic rats. Zinc (Zn) is an essential trace element in many enzymes and is involved in antioxidant defense. Numerous studies have shown that zinc supplementation has a protective effect against diabetic renal damage by promoting metallothionein synthesis (30, 31) and regulating oxidative stress (32–34). Meanwhile, triethylenetetramine (TETA) is a Copper (II)-selective chelation that inhibits copper-mediated oxidative stress and restores antioxidant defenses. A growing body of research suggests that TETA may reduce the chronic complications of diabetes by enhancing antioxidant defense mechanisms (35, 36).

Since copper and zinc are important components of superoxide dismutase (SOD), selenium is a component of the antioxidant enzyme GSH-Px, both of which are major natural antioxidants capable of scavenging excess free radicals from the body. Therefore, we speculate that renal function damage in spontaneous diabetic rats induces an increase in the excretion of urinary trace elements, which in turn causes trace element metabolism disorders. However, 1,25(OH)_2_D_3_ intervention may improve the metabolic disorder of trace elements by reducing the renal function damage in ZDF rats, among which copper, zinc, and selenium are most closely related to oxidative stress. Ito further pointed out that increased urinary copper excretion in patients with advanced diabetic nephropathy may be due to the copper-albumin and ceruloplasmin-copper complexes through the impaired glomeruli (37). Considering that the related mechanism of 1,25(OH)_2_D_3_ improving specific trace elements in urine is unknown, future research is warranted to explore the underlying mechanisms. Except for urine trace elements, we also focused the urinary levels of Ca and Mg. In brief, 1,25(OH)_2_D_3_ may accelerate urinary Ca and urinary Mg excretion in the early period, which is less pronounced with the extension of the intervention. This phenomenon needs to be further confirmed by other relevant studies.

Eventually, from our study, we speculate that 1,25(OH)_2_D_3_ supplementation may have a positive effect in diabetic rats that are deficient or inadequate in vitamin D status. Conversely, if vitamin D levels are adequate in diabetic rats, 1,25(OH)_2_D_3_ intervention may have no significant improvement. This is an important hypothesis that we need to validate in our next study.

## Conclusion

In summary, our research confirmed that vitamin D-deficient diabetic rats suffered from renal injury and disturbance of trace element metabolism, manifested by increased production of MALB, UACR, urine creatinine content, and urinary excretion of trace elements. Interestingly, we found that 1,25(OH)_2_D_3_ reversed renal impairment and improved urinary excretions of Cu, Zn, Se, and Mo in ZDF rats. Our study indicated that 1,25(OH)_2_D_3_ may be a potential therapeutic strategy for correcting trace element disorders in DN patients in the near future.

## Data availability statement

The original contributions presented in this study are included in the article/supplementary material, further inquiries can be directed to the corresponding authors.

## Ethics statement

This animal study was reviewed and approved by the Animal Ethical Committee of Tongji Medical College.

## Author contributions

DW: conceptualization, performing the experiment, and writing—original draft preparation. NW and JZ: performing the experiment. GLu: interpreting the results. YL: conceptualization. WY and HT: data analysis. GLi and JW: funding acquisition. LH: revising the manuscript. All authors contributed to the article and approved the submitted version.
